# Preliminary safety and effectiveness of psilocybin-assisted therapy in adults with fibromyalgia: an open-label pilot clinical trial

**DOI:** 10.3389/fpain.2025.1527783

**Published:** 2025-03-18

**Authors:** Jacob S. Aday, Jenna McAfee, Deirdre A. Conroy, Avinash Hosanagar, Vijay Tarnal, Cody Weston, Katherine Scott, Dana Horowitz, Jamarie Geller, Steven E. Harte, Niloufar Pouyan, Nicolas G. Glynos, Anne K. Baker, Jeffrey Guss, Alan K. Davis, Helen J. Burgess, George A. Mashour, Daniel J. Clauw, Kevin F. Boehnke

**Affiliations:** ^1^ Department of Anesthesiology, University of Michigan Medical School, Ann Arbor, MI, United States; ^2^ Michigan Psychedelic Center, University of Michigan, Ann Arbor, MI, United States; ^3^ Psychiatry Department, University of Michigan Medical School, Ann Arbor, MI, United States; ^4^ General Health Science, Veterans Affairs Ann Arbor Healthcare System, Ann Arbor, MI, United States; ^5^ Neuroscience Graduate Program, University of Michigan, Ann Arbor, MI, United States; ^6^ Department of Psychiatry, NYU Grossman School of Medicine, New York, NY, United States; ^7^ Center for Psychedelic Drug Research and Education, College of Social Work, The Ohio State University, Columbus, OH, United States; ^8^ Comprehensive Cancer Center, Department of Internal Medicine, Division of Medical Oncology, The Ohio State University, Columbus, OH, United States; ^9^ Center for Psychedelic and Consciousness Research, Department of Psychiatry and Behavioral Sciences, Johns Hopkins University, Baltimore, MD, United States

**Keywords:** psilocybin, psilocybin-assisted therapy, fibromyalgia, clinical trial, pilot

## Abstract

**Introduction:**

Fibromyalgia (FM) is the prototypical nociplastic pain condition, characterized by widespread pain and issues with cognition, mood, and sleep. Currently, there are limited treatment options available that effectively treat FM symptoms. Psilocybin-assisted therapy (PAT) is an emerging combined drug-therapy intervention, but no studies to-date have investigated PAT for FM.

**Methods:**

Here, we report findings from an open-label, pilot clinical trial of PAT for FM (*N* = 5). In conjunction with psychotherapy (two preparatory, four integration sessions), participants received two doses of oral psilocybin (15 mg and 25 mg) delivered two weeks apart.

**Results:**

Regarding safety (primary outcome), there were transient elevations of blood pressure or heart rate during dosing which normalized by the end of treatment, with no serious adverse events. Four of five participants reported transient headaches following dosing. Compared to baseline, participants reported clinically meaningful improvements in the following secondary outcomes one month following their second psilocybin dose (reported as Cohen's *d*): pain severity [*d* = −2.1, 95% CI(−3.7 to −0.49)], pain interference [*d* = −1.8, 95% CI (−3.27 to −0.24)], and sleep disturbance [*d* = −2.5, 95% CI (−4.21 to −0.75)]. Using the Patient Global Impression of Change, one participant reported their symptoms “very much improved,” two reported “much improved,” and two reported “minimally improved.” We stopped recruitment early because of concerns about generalizability and changes in FDA guidance for psychedelic clinical trials that occurred data collection.

**Discussion:**

This small open-label trial preliminarily supports that PAT is well-tolerated by people with FM, establishing a basis for larger randomized controlled trials.

**Clinical Trial Registration:**

ClinicalTrials.gov, identifier, (NCT05128162).

## 1 Introduction

Fibromyalgia (FM) is a common chronic pain condition that affects 2%–4% of the general population (1). FM is currently understood as the prototypical nociplastic pain condition, i.e., a disorder of pain regulation via altered centralized pain processing (2). Common FM symptoms include widespread pain, poor sleep quality, fatigue, and cognitive difficulties (3), and many individuals with FM have a history of trauma and/or comorbid psychiatric conditions (4). The heterogeneous nature of FM manifests clinically as varied phenotypic expressions, which makes many treatment options, such as pharmacotherapy and physical interventions, only minimally effective (5). This leads to considerable divergence in patient outcomes and results in significant economic and personal burden (6). Given the mixed success of existing treatments, there is a pressing need to develop tailored and more effective therapeutic approaches (7).

One potential treatment for FM is psilocybin-assisted therapy (PAT) (8), a treatment paradigm that combines psychotherapy with administration of psilocybin (9). Psilocybin is a serotonergic compound found in numerous mushroom species that causes substantial alterations in cognition, mood, affect, and sensory experience (10). Human studies with psilocybin indicate altered functional connectivity of neuronal networks associated with clinical benefits (11, 12). Speculatively, the subsequent changes in functional connectivity by psilocybin might also facilitate changes associated with clinical benefits in conditions characterized by nociplastic pain, but this remains untested. Additionally, psychedelic use and PAT have been linked with increased psychological flexibility (13, 14), and it hypothesized that insights gained during PAT therapy sessions may be more likely to “stick” and facilitate lasting psychological and behavioral change, particularly in disorders characterized by psychological rigidity (15–17). Indeed, recent studies of PAT have demonstrated promising findings for treatment of major and treatment-resistant depression (18–21), anxiety (20–23), end-of-life distress (24, 25), obsessive compulsive disorder (26), and substance use disorders (27, 28).

Preliminary clinical trials and case studies suggest that psilocybin may produce analgesic effects in intractable phantom limb pain (29), cluster headache (30, 31), and migraine (32). This has been complemented by recent preclinical research demonstrating that single-dose psilocybin reduced mechanical hypersensitivity in rats for at least 28 days (33). Similarly, surveys show that many individuals using psilocybin and other psychedelics for chronic pain report substantial pain relief (34–36). In a North American survey study of participants with fibromyalgia (37), perceptions of benefit from psychedelic use were generally neutral (59.4%) or positive (36.8%), with less than 3% reporting negative impacts on overall health or pain symptoms. Notably, of the 12 participants in that study who reported using psychedelics with the specific intention of treating chronic pain, 11 reported improved symptoms.

In summary, converging lines of research support the mechanistic potential of PAT in the treatment of FM. However, clinical studies assessing the safety and tolerability of psilocybin and associated therapy in people with FM are limited. Thus, in the current pilot study, we evaluated the preliminary safety and effectiveness of PAT in the treatment of FM using an open-label design among five individuals.

## 2 Materials and methods

This open-label clinical trial was conducted at the University of Michigan Chronic Pain and Fatigue Center in Ann Arbor, Michigan. All study procedures were approved by the University of Michigan Medical School Institutional Review Board under protocol HUM00208367. The study was prospectively registered on clinicaltrials.gov under identifier NCT05128162. The study protocol is available in Supplementary 1. In addition to the study procedures outlined below, participants also completed phenotyping visits consisting of sensory testing and magnetic resonance imaging, the results of which will be reported separately.

### 2.1 Study design and participants

**Figure 1:**
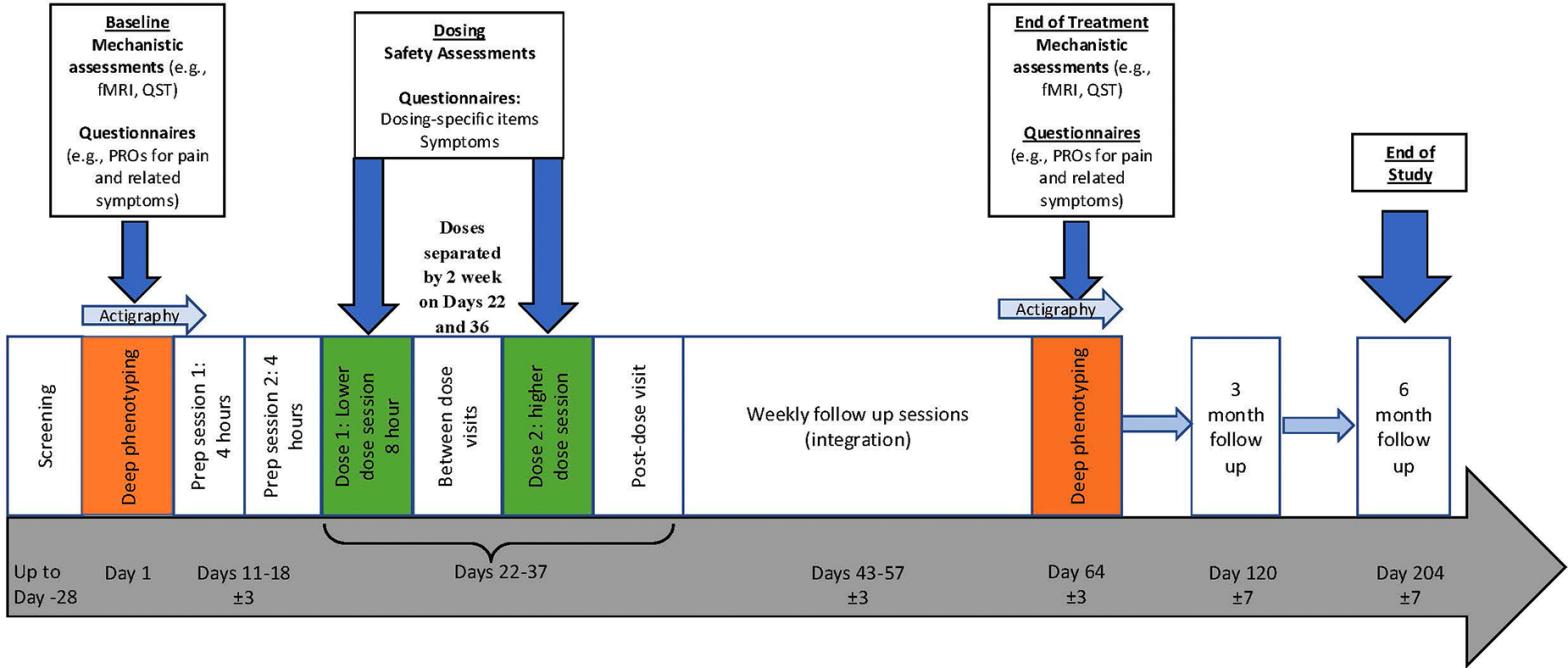
Overall study design.

Participants in this trial were adults aged 25–64 who did not smoke tobacco and had a diagnosis of FM or who had reported FM symptoms for the past year (Figure 1). At screening, all participants also met the 2016 FM survey criteria for fibromyalgia (38). Exclusion criteria included being pregnant or nursing, cardiovascular condition in the past year (e.g., coronary artery disease, stroke, angina, uncontrolled hypertension, transient ischemic attack), epilepsy, insulin-dependent diabetes, active autoimmune disease, clinically significant laboratory abnormalities per a complete blood count and metabolic panel, past year or current substance use disorder (other than caffeine), past or current history of having a psychotic disorder or bipolar I or II disorder. All screened subjects’ (*n* = 17) medical records were reviewed by study physicians and those found to be eligible after medical record review met with study physicians to ensure they did not meet any exclusionary medical criteria. Participants with scores representing severe depression on the Patient Health Questionnaire 8 were also excluded (39). Exclusionary medications included monoamine oxidase inhibitors, psychoactive prescription medications (e.g., benzodiazepines, opioids) more than twice per week, prohibited drugs of abuse including illicit opioids, cocaine, methamphetamines, 3,4-Methyl enedioxy methamphetamine, and any use of hallucinogens in the past 6 months or more than 10 lifetime hallucinogen uses. Cannabis was allowed if it was part of the participant's treatment regimen prior to enrollment. Other prohibited medications included antihypertensive medications, UGT1A9 or 1A10 inhibitors (e.g., regorafenib, rifampicin, deferasiroxor, ginseng) and aldehyde or alcohol dehydrogenase inhibitor (e.g, disulfiram). Initially, participants taking antidepressant medications of any variety were excluded, but following recent studies showing the safety of concomitant antidepressant use during PAT (40), we amended our protocol to include individuals taking selective serotonin reuptake inhibitors (SSRIs) and selective norepinephrine reuptake inhibitors (SNRIs) as well as less than 300 mg/day of bupropion. A full description of eligibility criteria are included in the study protocol. Participants were recruited through flyers posted in University of Michigan clinics, UMhealthresearch.org, and electronic health record searches with IRB permission between September 2023 and April 2024.

### 2.2 Description of therapy

Therapy visits occurred at the Sleep and Circadian Research Laboratory and via Zoom (certain therapy visits only). The therapeutic approach used in this study was informed by “The Yale manual for psilocybin-assisted therapy of depression (using acceptance and commitment therapy as a therapeutic frame)”, an established theoretical framework for PAT (41). However, the manual was modified for fibromyalgia with input from Fluence, a continuing education organization in the emerging field of PAT, as well as consultation with experts in delivering PAT (co-authors Drs. Davis and Guss). Each participant was paired with a consistent therapist dyad throughout the study, consisting of a lead therapist and co-therapist. The lead therapists were doctoral level psychotherapists (JM and DAC) and the co-therapist had a Masters of Social Work (DH).

As is standard in PAT research, the therapy component included preparatory, dosing, and integration sessions with the therapist dyad (42). During the preparatory sessions, the therapists (a) developed a trusting therapeutic rapport with participants, (b) gathered information about lived experience with FM, (c) provided education regarding psychedelic experiences, (d) described the therapeutic approach to be used, and (e) explored participants’ expectations for treatment. Therapists explained the logistics of the dosing sessions (e.g., dosing day procedures, session duration, music, use of eyeshades), delineated behavioral boundaries of interaction between the participant and the therapists, and discussed safety measures. In dosing sessions, therapists maintained an attentive but non-intrusive presence, encouraging participants to focus inward on their thoughts, emotions, and body sensations, including engaging with difficult content that arose. They also assisted the participant by meeting any immediate needs for comfort or safety. Toward the end of the dosing sessions, participants were invited to talk about their experiences in the session and the emotions that were evoked. Therapists focused on eliciting descriptions of phenomenology, rather than interpreting or guiding this report. The integration phase began the day after the dosing sessions and involved thoroughly reviewing the participant's experience during the dosing session and, in some cases, reinforcing aspects of the experience to foster changes in thought and behavior. Therapists accomplished this by asking open-ended questions about the session, intended to elicit introspective, interpersonal, spiritual, or noetic insights that occurred during the session that the participant may otherwise forget or have difficulty verbalizing. Therapists supported the participants’ narrative expression of their experience and emphasized this over interpretive interventions.

### 2.3 Study drug

Study drug was provided as psilocybin in 15 mg and 25 mg gel capsules synthesized by the analytical chemistry lab at the Usona Institute. Participants received two doses of psilocybin (15 mg followed by 25 mg) approximately two weeks apart. Sequential dosing was chosen in alignment with previous studies (19, 43), as well as to allow participants to acclimate to the pharmacodynamic effects of psilocybin in the first dose and thus to prepare for the more intense effects in the second dosing session. Participants could decide to not increase their dose to 25 mg at the second dosing session, and one participant (001) chose not to do so. 25 mg was selected as the high dose based on research that demonstrates tight dose-response curves across studies of various indications, which are supplemented by findings that body mass index (44) and weight (45) are not significantly related to drug effects.

### 2.4 Dosing days

For 7 days prior to dosing sessions, participants agreed to abstain from taking nonprescription medications, nutritional supplements, or herbal supplements except when approved by the study team. Similarly, they agreed to abstain from alcoholic beverages and psychoactive drugs 24 h before and after each dosing session, and sildenafil within 72 h before or after dosing days. On the day of drug administration, participants were asked to eat a low-fat breakfast before reporting to the laboratory. Upon arrival, participants provided urine samples to test for exclusionary drugs and pregnancy and completed breath alcohol and COVID-19 tests. Study therapists also underwent COVID-19 testing on dosing days. If all tests were negative, participants met with the study team for a brief interview to discuss if the session was contraindicated. After review of their vital signs and clearance from the study physician, participants then proceeded to a comfortable dosing suite with the same therapists that were present during preparatory sessions. The dosing suite included a living room-like space with a couch for the participant and two armchairs for the therapists as well as an adjacent bedroom where the participants could lie down if they preferred and a bathroom. During these sessions, participants were instructed to lie on the couch or bed, wear eyeshades, and listen to music through headphones or a headband, all of which are meant to enhance and encourage internal attention and reflection. The same playlist, created by researchers at The Ohio State University, was used for every dosing session. Vitals (i.e., heart rate and blood pressure) were intermittently monitored throughout the 8-hour dosing sessions (30, 60, 90, 120, 180, 240, 300, and 360 min after administration). During the same period as the vital signs measurements, therapists also completed a monitor rating form, which included questions about the presence/intensity of behaviors, signs, and reported symptoms, such as peacefulness, yawning, nausea/vomiting, quantity of speech, anxiety, sleepiness, crying, restlessness, visual changes, euphoria, and feelings of unreality. Study physicians examined the participant if any concerns were raised by study therapists or if there was a concern for elevated vitals. Dosing sessions were video and audio recorded. After the study drug effects subsided, participants completed questionnaires that assessed the subjective experiences of the dosing session (e.g., *Mystical Experience Questionnaire, Challenging Experiences Questionnaire*), and the therapists completed questionnaires assessing participant mood and safety. Participants were medically cleared by the study team (including a study physician) and were discharged home with a responsible adult. Participants were asked to process the experience at home by writing a reflection about their experiences during the dosing session and this reflection was discussed during follow-up integration sessions.

### 2.5 Measures

#### 2.5.1 Primary outcome

The primary outcome for this study was safety. This was assessed acutely during dosing sessions via heart rate per minute and blood pressure, as well as globally via adverse event capture from Days 1 (baseline) through 64 (end of treatment). Blood pressure greater than 200 systolic or greater than 110 diastolic for more than 15 min was considered to be an adverse event. For heart rate, our goal was to maintain a target heart rate within 20% of baseline. An elevated heart rate was considered clinically significant if it was accompanied by cardiovascular symptoms and an increase in blood pressure (exceeding 20% of baseline) that persisted for over 15 min.

#### 2.5.2 Secondary outcomes

##### 2.5.2.1 Aggregate worst pain score change

We assessed participants’ worst pain intensity daily using a 0–10 (0 = no pain, 10 = worst pain imaginable) numeric rating scale via a daily Qualtrics survey. We compared the average of pain scores from Days 1 to 7 (prior to preparation therapy) to the average of pain scores from the 7-day window (Days 57–63) immediately prior to the end of treatment visit (Day 64).

##### 2.5.2.2 Pain interference

Pain interference is the degree to which pain affects important aspects of an individual's life, such as social, cognitive, and physical activities. We assessed pain interference using the 4-item PROMIS pain interference scale from the PROMIS-29 + 2 Profile v2.1 (PROPr) (46, 47). This uses a 5-point Likert scale to generate raw scores. We then used the HealthMeasures Scoring Service to generate standardized T-scores, which range from 0 to 100 (mean = 50, which represents the general population mean, standard deviation = 10). Higher scores indicate worse pain interference.

##### 2.5.2.3 Sleep disturbance

Sleep disturbance includes subjective assessment of sleep quality, perceived ability to fall and stay asleep, satisfaction of sleep, and depth of sleep. We assessed sleep disturbance using the PROMIS Sleep Disturbance Short Form 8b. Scoring was performed the same way as for pain interference, with standardized t-scores ranging from 0 to 100 (mean = 50, which represents the general population mean, standard deviation = 10). Higher scores indicate worse sleep disturbance.

##### 2.5.2.4 Chronic pain acceptance

The Chronic Pain Acceptance Questionnaire (8 item version) is a validated measure that assesses activity engagement and pain willingness, e.g., recognizing that trying to avoid or control pain may be maladaptive for chronic pain (48). Participants rate items on a 0–6 scale, with 0 being “never true” and 6 being “always true”. Higher scores indicate higher acceptance of chronic pain.

##### 2.5.2.5 Patient global impression of change (PGIC)

We assessed participant impressions of how PAT affected their global functioning using the PGIC, a one-item questionnaire that uses a 7-point Likert scale ranging from 1 (“very much improved”) to 7 (“very much worse”) (49–51).

#### 2.5.3 Exploratory outcomes

##### 2.5.3.1 Challenging experiences questionnaire

The *Challenging Experiences Questionnaire* (*CEQ*)is a 26-item self-report survey that was used to assess the extent to which participants endorsed having psychologically challenging experiences during their dosing sessions (52). The survey includes seven factors: grief, fear, death, insanity, isolation, physical distress, and paranoia.

##### 2.5.3.2 Mystical experiences questionnaire

The *Mystical Experiences Questionnaire* (*MEQ30*) is a 30-item questionnaire that is commonly used to index mystical-type experiences (i.e., experiences characterized by a sense of transcendence of time and space, unity, ineffability, and deep meaning) induced with psychedelics and other altered states of consciousness (53). The survey includes four facets: “mystical”, “positive mood”, “transcendence”, and “ineffability”.

##### 2.5.3.3 Other psychosocial functioning

We assessed anxiety, depression, fatigue, participation in social activities, and cognitive abilities, using the scales from the PROMIS-29 + 2. Scoring was conducted in the same way as for pain interference and sleep disturbance, with standardized t-scores for each subscale ranging from 0 to 100 (mean = 50, standard deviation = 10). Higher scores indicate worse symptoms for pain severity, pain interference, sleep disturbance, anxiety, depression, fatigue, and cognitive abilities. Lower scores indicate worse symptoms for physical function and participation in social activities. Due to a coding error, the data on participation in social activities was not usable so will not be reported in this manuscript.

##### 2.5.3.4 Narrative of dosing experiences

At the end of each dosing session (Visits 5 and 8), study therapists asked participants to write a narrative describing their experience of the sessions prior to their next in-person meeting. This narrative description was discussed in Visits 6 and 9, respectively, and used as content to draw from in the integration sessions. We present summaries of how each participant experienced psilocybin dosing sessions, drawing directly from the content of their narratives.

### 2.6 Statistical analysis

We present descriptive statistics for primary and secondary outcomes. We present measures of effect sizes (baseline vs. end of treatment) as Cohen's *d* with 95% confidence intervals for secondary outcomes (sleep disturbance, chronic pain acceptance, pain severity, pain interference) and exploratory outcomes (physical function, anxiety, depression, fatigue, cognitive abilities). For the Patient Global Impression of Change, we present the participant ratings at the end of treatment. Results are presented for each individual participant as well for participants in aggregate. Two researchers (JSA & KFB) individually reviewed participants’ written narratives of the dosing sessions and each generated a list of prominent themes and quotes. They then compared thematic categories and generated a combined set of themes for each dosing session. These themes and quotes were distilled into brief narratives for each participant included below.

## 3 Results

**Figure 2:**
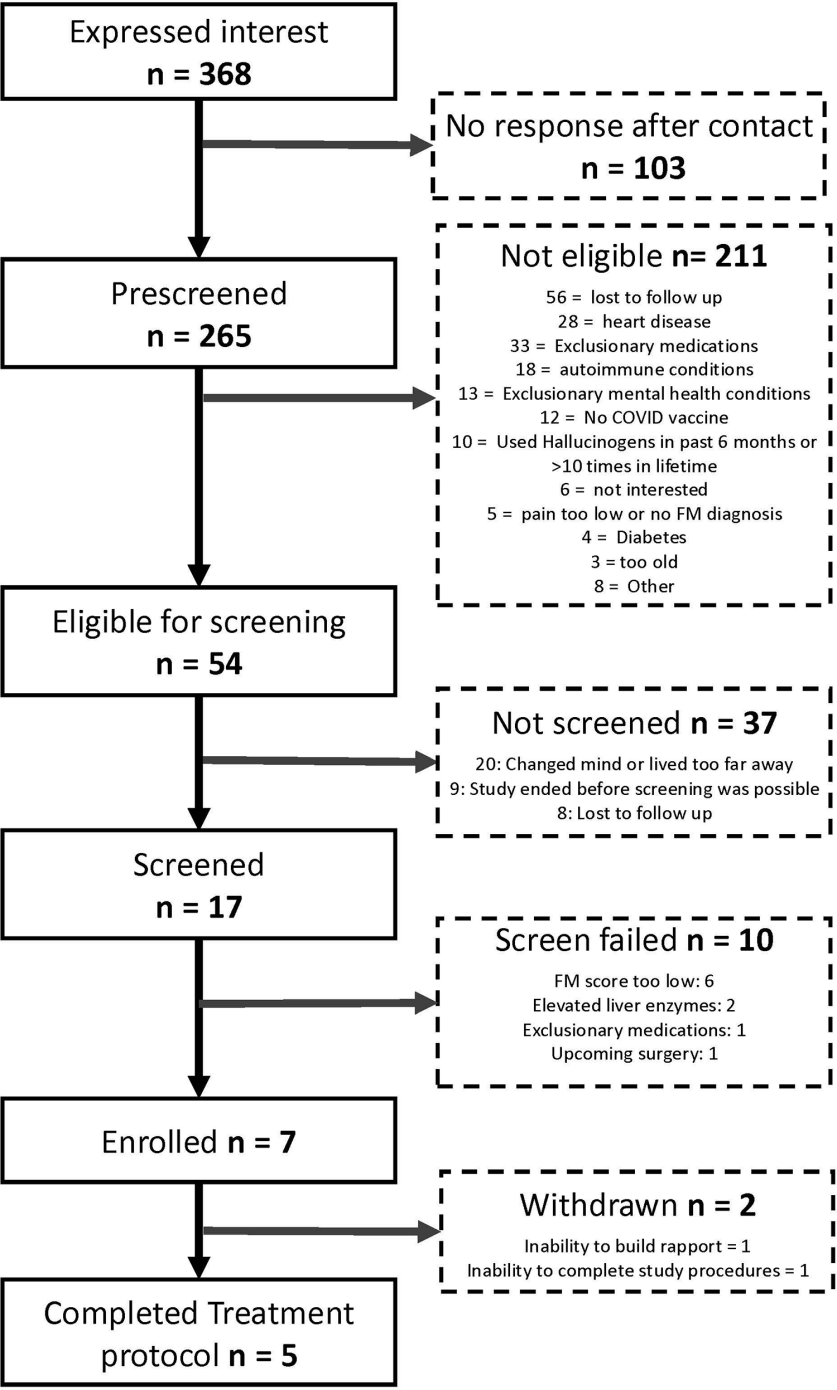
CONSORT diagram.

Overall, 368 individuals expressed interest in the study, 265 were pre-screened, 54 were eligible for screening, 17 were brought in for screening, and 7 were eligible for participation (Figure 2). Two participants were withdrawn by the study team after consenting given concerns about ability to follow through with study procedures (*n* = 1) and concern about their ability to build rapport with study therapists (*n* = 1). Our original recruitment target was *n* = 10 individuals, but we stopped recruitment early due to challenges with recruitment, concerns about the generalizability of our results given the stringent exclusion criteria, and that standards for psychedelic research changed during the conduct of our trial following the US Food and Drug Administration's publication of guidance for psychedelic clinical trials (54). For example, the more recent guidance have: (1) loosened requirements for exclusionary psychiatric conditions (e.g., bipolar type II) (55), (2) suggested that the lead monitor during dosing should not be present during follow-up psychotherapy, and (3) previous exclusionary medications per the FDA process are no longer exclusionary (e.g., selective serotonin reuptake inhibitors) (54). Some contemporaneous studies even allow there to be treatment in the absence of psychotherapy but with psychological support (18).

Of the *n* = 5 participants who completed the protocol, all were female, four identified as White/Caucasian, and one identified as Black/African American (Table 1).

**Table 1:** Participant demographics and baseline clinical characteristics.

Measures	Participants				
	Participant 001	Participant 002	Participant 003	Participant 004	Participant 005
Age (years)	63	41	46	62	37
Sex at birth	Female	Female	Female	Female	Female
Race	Black African Amer	White	White	White	White
Highest education achieved	Bachelor's	Bachelor's	Advanced/Professional	Bachelor's	Advanced/Professional
Employment status	Retired	Full time employed	Full time employed	Retired	Marked Unemployed—wrote self-employed
Previous psychedelic use	No	No	Yes	No	Yes
Current cannabis use (confirmed by urinalysis)	Yes	No	Yes	Yes	No
Clinical symptoms					
Pain severity	5.9	4.0	5.4	5.4	3.7
Chronic pain acceptance	31.0	31.0	25.0	32.0	23.0
Sleep disturbance	58.3	55.3	62.6	58.3	56.3
Pain interference	66.6	57.1	63.8	63.8	61.2
Physical function	36.7	48.3	40.5	40.5	41.9
Anxiety	51.2	55.8	55.8	48.0	55.8
Depression	41.0	41.0	51.8	41.0	53.9
Fatigue	58.8	53.1	64.6	64.6	62.7
Cognitive abilities	44.3	50.5	44.3	44.3	44.3
FM survey score	17.0	11.0	19.0	14.0	12.0

Pain severity: 0–10 numeric rating scale. Sleep disturbance, pain interference, physical function, anxiety, depression, fatigue, cognitive abilities, and participation in social activities are reported as T-scores, with 50 being the general population mean. Most participants had substantially worse sleep, pain interference, physical function, fatigue, cognitive abilities, and participation in social activities than the population mean.

### 3.1 Safety

**Figure 3:**
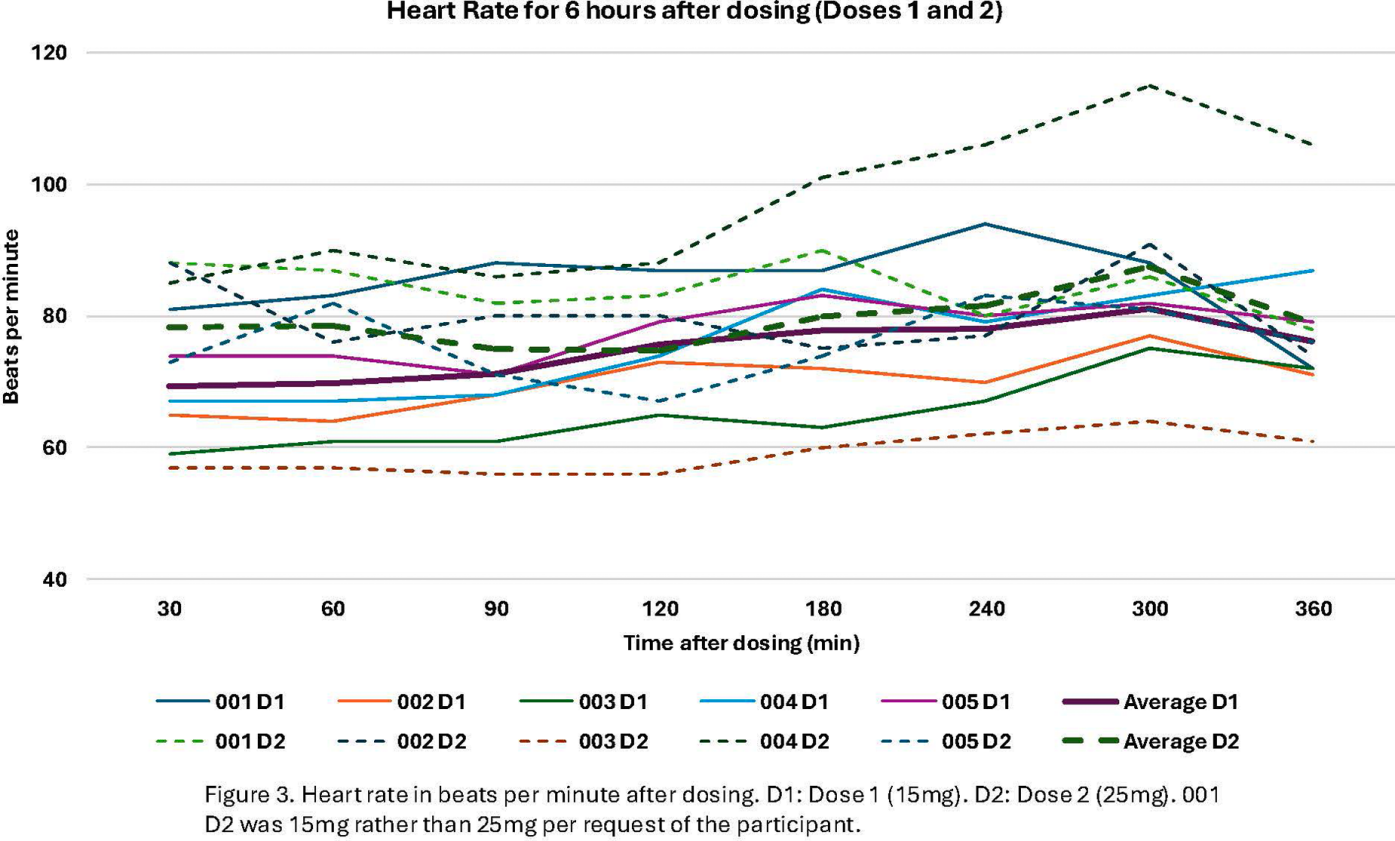
Heart rate in beats per minute after dosing. D1: Dose 1 (15 mg). D2: Dose 2 (25 mg). 001 D2 was 15 mg rather than 25 mg per request of the participant.

**Figure 4:**
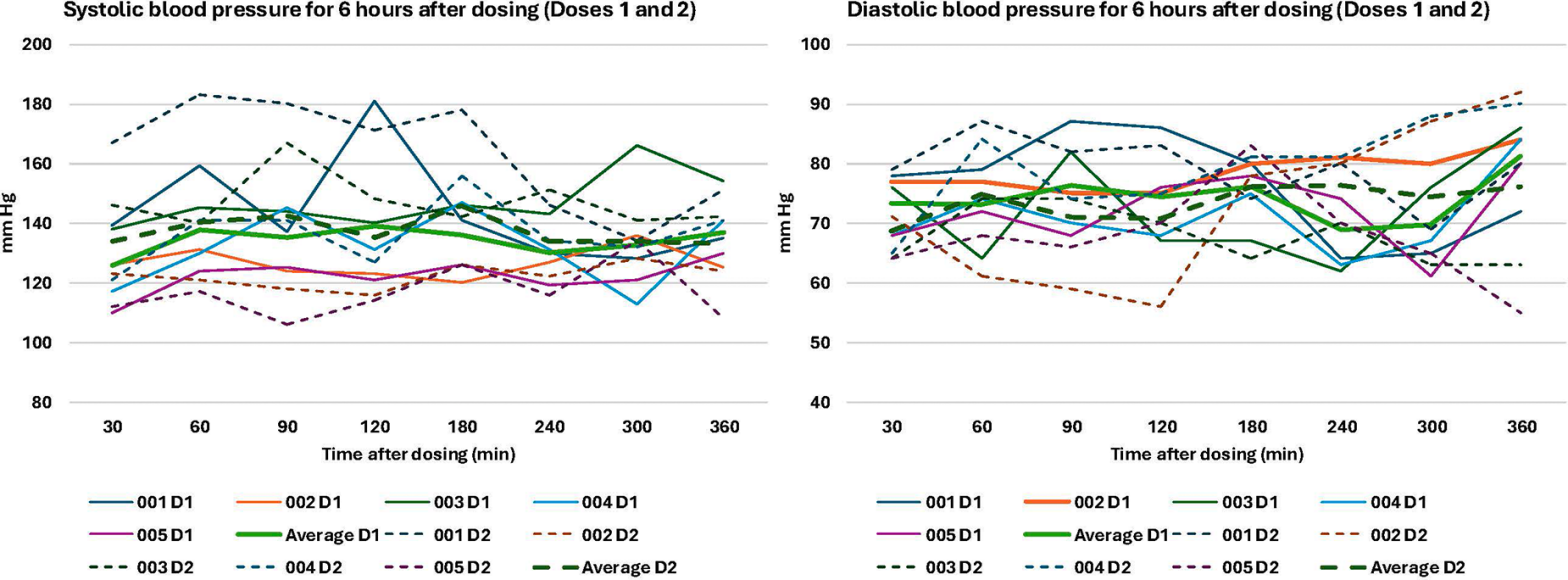
Systolic and diastolic blood pressure after doses 1 and 2. D1: Dose 1 (15 mg). D2: Dose 2 (25 mg). If a participant had blood pressure >200 systolic or >110 diastolic for >15 min, they would be transferred to the emergency department.

Overall, the dosing scheme in this protocol was well-tolerated, with no serious adverse events (Table 2). Participant 001 opted to have their second dose be 15 mg rather than 25 mg, with their rationale being that they thought the 15 mg dose was sufficient and they wanted to experience it again. AEs related to the study treatment occurred on or the day after dosing and were deemed minor or moderate, with the most common being headache (*n* = 4), diarrhea (*n* = 2), stomach ache (*n* = 1), and migraine (*n* = 1). The headaches were of minor or moderate severity, and three participants took over the counter pain medications (acetaminophen, celecoxib) to help relieve headaches. Headaches resolved within two days. Blood pressure and heart rate stayed within the pre-specified ranges during dosing and were not associated with any other cardiovascular symptoms (Figures 3, 4). Results from the *Challenging Experiences Questionnaire* are presented in Table 3. Physical distress and grief were the most commonly reported domains of challenging experiences. Results from the *Mystical Experiences Questionnaire* are included in Table 4. 3 out of 5 participants had a “complete” mystical experience (i.e., >60% on all facets) (53) during the first dose and 4 out of 5 participants had a mystical experience during the second dose.

**Table 2:** Adverse events prior, during, and after dosing.

Adverse events	Study visit		
	V2-V4 (preparation)	V5-V9 (administration)	V10-V13 (integration)
Adverse event (AE)^a^	5	11	8
Related AE^b^	0 (5)	4 (5)	0 (5)
Serious AE	0 (5)	0 (5)	0 (5)
AE severity for related			
Mild	0 (5)	4 (5)	0 (5)
Moderate	0 (5)	1 (5)	0 (5)
Severe	0 (5)	0 (5)	0 (5)
AE type			
Headache	1 (5)	4 (5)	0 (5)
Diarrhea	0 (5)	2 (5)	1 (5)
Stomach ache	0 (5)	1 (5)	0 (5)
Migraine	0 (5)	1 (5)	0 (5)
Leg throbbing	0 (5)	0 (5)	1 (5)
Back ache	1 (5)	1 (5)	0 (5)
Jaw pain	1 (5)	1 (5)	0 (5)
Fatigue	1 (5)	0 (5)	0 (5)
Sore throat	0 (5)	0 (5)	1 (5)
Sinus pressure	0 (5)	0 (5)	1 (5)
Vomiting	0 (5)	0 (5)	1 (5)
Fever	0 (5)	0 (5)	1 (5)
Chills	0 (5)	0 (5)	1 (5)
Body aches	0 (5)	0 (5)	1 (5)
Tight L side of neck	1 (5)	1 (5)	0 (5)
Medication taken for related AE	0 (5)	3 (5)	0 (5)

AE, adverse event.

^a^ Participants are counted once for each category regardless of the number of events.

^b^ Related AE: An AE was classified as “related” if there was a reasonable possibility that the study drug or procedure caused the event. Severity and relationship to study drug or procedure were determined by the Principal Investigator. Participant 001 repeated the 15 mg dose at 2nd dosing session. All other participants had the 15 mg followed by the 25 mg dose.

**Table 3:** Challenging experiences questionnaire (%).

Participant	Fear	Grief	Physical distress	Insanity	Isolation	Death	Paranoia	CEQ total
	(Dose 1, dose 2)	(Dose 1, dose 2)	(Dose 1, dose 2)	(Dose 1, dose 2)	(Dose 1, dose 2)	(Dose 1, dose 2)	(Dose 1, dose 2)	(Dose 1, dose 2)
001	0%, 0%	50%, 27%	0%, 0%	0%, 0%	0%, 0%	0%, 0%	0%, 0%	12%, 6%
002	20%, 20%	17%, 13%	88%, 76%	0%, 0%	0%, 0%	0%, 0%	0%, 0%	25%, 22%
003	0%, 16%	60%, 37%	20%, 48%	0%, 20%	47%, 20%	0%, 80%	30%, 0%	25%, 32%
004	0%, 12%	0%, 47%	64%, 60%	0%, 0%	0%, 40%	0%, 0%	0%, 0%	12%, 29%
005	12%, 0%	0%, 0%	28%, 16%	0%, 0%	0%, 0%	0%, 0%	0%, 0%	8%, 18%

Participant 001 received 15 mg at both dosing sessions.

**Table 4:** Mystical experiences questionnaire (%).

Dose 1	Participant	Mystical	Positive mood	Transcendence	Ineffability	Total	Complete mystical experience
	001	96%	86%	64%	89%	84%	Yes
	002	33%	44%	53%	33%	41%	No
	003	61%	64%	64%	61%	63%	Yes
	004	76%	89%	94%	89%	87%	Yes
	005	51%	61%	47%	67%	57%	No
Dose 2							
	001	94%	78%	78%	89%	85%	Yes
	002	26%	50%	42%	50%	42%	No
	003	83%	78%	81%	78%	80%	Yes
	004	68%	83%	97%	83%	83%	Yes
	005	89%	78%	72%	83%	81%	Yes

Participant 001 received 15 mg at both dosing sessions.

### 3.2 Preliminary effectiveness

**Figure 5:**
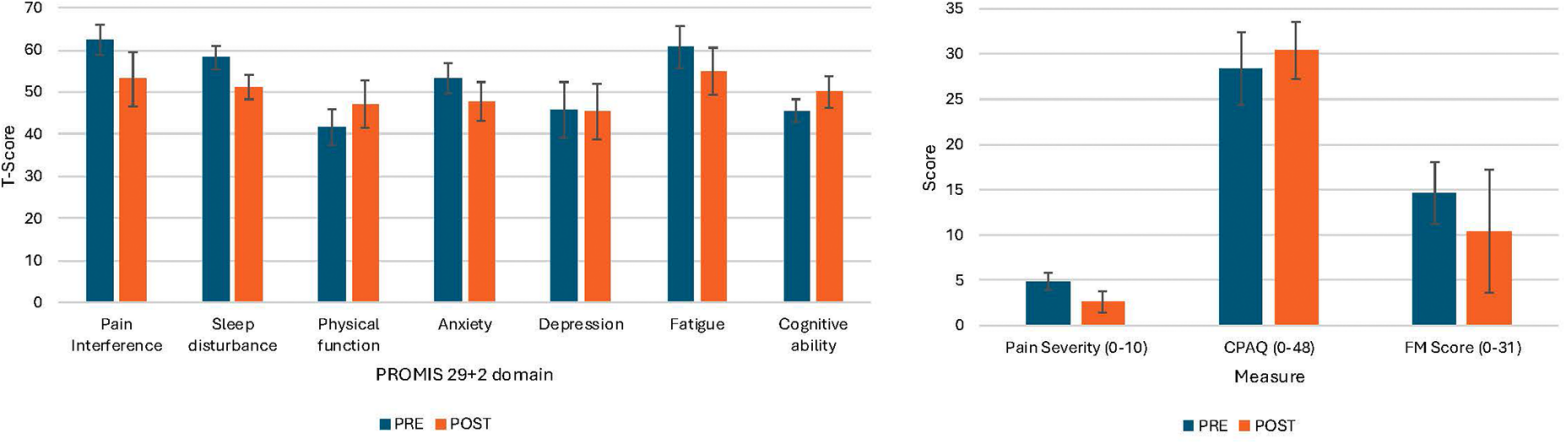
CPAQ: chronic pain acceptance questionnaire. Pain Severity reported as change in aggregate pain score from the 7 days prior to the intervention to the end of the intervention. Sleep disturbance, pain interference, physical function, anxiety, depression, fatigue, participation in social activities, and cognitive abilities are all reported as T-scores per PROMIS scoring. Negative change scores indicate improvement for pain severity, pain interference, sleep disturbance, FM score, anxiety, depression, and fatigue. Positive change scores indicate improvement for CPAQ, physical function, participation in social activities, and cognitive abilities.

Some participants reported clinically significant improvements across numerous symptom domains. Figure 5 shows changes by individual as well as on average for each symptom domain. For secondary outcomes, three of five participants reported >2 point decrease in pain severity, four reported >4 point decreases in pain interference, and 4 reported >6 point decreases in sleep disturbance. Three participants reported enhanced chronic pain acceptance while two reported slight decreases in chronic pain acceptance. On average, there was a 2.3 ± 1.3 point decrease in pain severity [*d* = −2.1, 95% CI (−3.7 to −0.49)], 7 ± 4.2 point decrease in sleep disturbance [*d* = −2.5, 95% CI (−4.21 to −0.75)], 9.4 ± 4.2 point decrease in pain interference [*d* = −1.8, 95% CI (−3.27 to −0.24)], and 2 ± 2.8 point increase in chronic pain acceptance [*d* = 0.54, 95% CI (−74–1.79)]. Via the PGIC, one participant (001) reported that their symptoms were “very much improved,” two reported “much improved” (002, 004), and two reported “minimally improved” (003, 005). On average, FM survey scores improved by 4.2 ± 6.1 points [*d* = −0.78, 95% CI (−2.06–0.53)].

For exploratory outcomes (Figure 5), four of five participants reported decreases in anxiety, physical function, and fatigue, and three reported improvements in cognitive abilities. On average, there was a 5.4 ± 8.8 point increase in physical function [*d* = 2.1, 95% CI (−0.29–2.40)], 4.6 ± 4.5 point increase in cognitive abilities [*d* = 1.39, 95% CI (−0.05–2.77)], a 5.9 ± 3.7 point decrease in fatigue [*d* = −1.1, 95% CI (−2.46–0.25)], and a 5.6 ± 4.1 point decrease in anxiety [*d* = −1.4, 95% CI (−2.75–0.06)]. There were no substantial changes in depression scores [*d* = 0.03, 95% CI (−1.27–1.21)].

### 3.3 Qualitative narratives

Participant 001 reported strong feelings of connectedness with her family as well as dreamlike experiences during the first dosing session (15 mg) that included “time travel to ancient Egypt.” She also practiced yoga during this dosing session. During the second dosing session (also 15 mg), this sense of connectedness extended to messaging with a forest and stars, with the forest saying, “We're in trouble. Humans are destroying us. We are all connected.” and the stars pointing to the “origin of original injury,” which was a car accident when the participant was 13 years old. Following this, the participant noted that these interactions gave her insight on how to “repair” her body so she “started moving & doing yoga.”

In contrast to 001, Participant 002 did not have a pleasant experience for either dose, and was “disappointed it was not a more enjoyable time”. She “felt almost as if I was not a good participant simply for having to use the bathroom” or removing headphones and the eyemask, tying into a realization that she had high expectations of herself and others that were not often met. Participant 002 also reported moderate pain during both sessions, including back pain during the first dosing sessions and that she “felt muscle spasms and parts of my body throb” during the second. Despite these negative experiences, she noted that the “medicine had numbed me” so the pain felt more manageable than it otherwise did. Although she experienced “no grand visions or revelations” and a headache like the “worst hangover” after the session, she believed “the medicine does help so its [sic] worth a day of being uncomfortable”.

The first dosing session for Participant 003 began with “slight visual distortions” and reflections on “lost loved ones, lost family members, things, places, people I don't think of often.” Early in the session, the participant was “surprised I've no pain to report” but experienced sweating throughout much of the day. At the end of session, she reported to be “awake, present, tired, limited pain, but altogether in a good place.” Themes of connection and a focus on loved ones continued in the second dosing session. The participant noted “I met my grandparents and great aunt again and gained such a sense of pride and love, I felt my grandmother's touch, as clear as can be.” This second dosing session had stronger visual components, including visiting a “shark habitat,” traveling “back to the caves, tunnels, and trees,” and a brief encounter with an “Asian red dragon” while “in the sky.” Another prominent theme seemed to be a generalized sense of gratitude: “Gratitude keeps surfacing—grateful for the journey, the time and space to rest, even gratitude for this often challenging body, since it means I'm still alive.”

Participant 004 noted powerful imagery in the first experience that involved moving spatially between different locations, such as moving from a lake into a forest, being “lifted to the sky to dance”, and then being in a cave where she could see the Northern Lights through a crevice. She was “physically comfortable” and was able to “push the music down my body to relieve the pain”. By the end of the experience, she was “warm, cozy and almost pain free”. The second experience also involved spatial movement between locations, such as moving from a hammock on a porch, to sitting at a kitchen table with family, then moving to a temple. Her reflections touched on constructing a narrative around her challenges in a way that suited her, noting “trauma has a negative connotation but for me it's been useful and even beautiful”. She also “acquired… the skill of “toughness””, reflecting that “I hold it, I have used it well” despite not wanting to have acquired it in the first place.

Participant 005 began by noting that “I had little expectations of what would come from this as far as long-term progress reduction of pain [sic]”. Their first dosing session was characterized by increased mental imagery, mindfulness (i.e., present moment awareness), and a sense of creativity. The participant was particularly engaged with the visual effects of the drug, often finding symbolism and beauty in their visions. They reported a lack of focus the day after dosing, feeling sadness and rejection “around certain issues”, and also sadness because she did not feel like doing the write-up and was worried it would not be good enough. The participant's second dosing session included a strong focus on introspection and connection to family, ancestors, and nature. They reported insights on familial relationships and on how to let her pain go and cope. They came to the conclusion that much of their pain stemmed from ancestral trauma and that it “felt really good knowing the pain is not mine. It's my ancestors but they're [sic] there's something that I can do about it for me and them.”

## 4 Discussion

This open-label investigation of PAT for fibromyalgia demonstrated that PAT was well-tolerated, with no serious adverse events associated with the study treatment. Mild and moderate AEs, such as headaches or diarrhea on the day of or after dosing, resolved shortly thereafter. There were no persistent psychological AEs by the end of treatment. Most participants also reported improvements in pain severity, pain interference, and sleep disturbance, with small improvements in chronic pain acceptance in three participants and a decrease in chronic pain acceptance in two. The improvements in secondary outcomes of pain severity, pain interference, sleep disturbance, and PGIC all align with what are considered clinically meaningful changes for these domains (51, 56–58).

Although there have been limited clinical trials of PAT for chronic pain conditions, our findings align with other preliminary work supporting analgesic effects of psilocybin. For instance, a small exploratory study in *n* = 16 participants with cluster headaches found that cluster attack frequency was significantly reduced in the three weeks following administration of psilocybin compared with placebo (*d* = 0.69) (30). Similarly, a proof-of-concept, double blinded, randomized, placebo controlled trial of migraine (*n* = 10) reported that the reduction in weekly migraine days from baseline was significantly greater after psilocybin than placebo (32). Lastly, a small open-label clinical trial in chronic cluster headache (*n* = 10) reported a 31% decrease in attack frequency from baseline-to follow-up 4 weeks after dosing was completed (31). Our findings also align with results from naturalistic studies of people using psilocybin for chronic pain (59). Survey studies have reported that a number of people using psilocybin and other psychedelics for a range of chronic pain conditions often report decreased pain symptoms following psychedelic use, and that in some cases psychedelics are more effective than their other pain medications (34, 60).

In addition to improvements in pain symptoms, participants also reported positive changes in mood, social cognition, sleep, and global functioning. These findings complement findings from the past decade indicating transdiagnostic potential of PAT for a variety of clinical conditions (61) as well as broad improvements in wellbeing observed when psychedelics are studied in healthy adults (62). For example, numerous studies have noted robust decreases in depression (18, 43) and anxiety following PAT (63). Although most participants in the current study reported decreases in anxiety, only one had a decrease in depression. This could possibly be driven by floor effects, given that the sample had below average depression scores at baseline because we excluded participants with severe depression. Similar to the current study, improvements in sleep have been observed in studies with psilocybin (64) and other psychedelics (65). Lastly, all participants reported at least some improvements in global functioning (e.g., one reported “very much improved”, two reported “much improved”, and two reported “minimally improved”). In summary, our preliminary findings motivate future research into the effects of PAT on the multimodal underpinnings of FM, including pain, mood, and sleep.

There were also numerous themes that emerged from participants’ reports of the dosing sessions that are consistent with those reported in previous research. First, most participants reported experiencing dreamlike states and vivid mental imagery that at times were considered to be highly symbolic and meaningful. This aligns with the well-established perceptual effects of the drugs (66) as well as recent findings that the visual effects of psilocybin and other psychedelics may play a role in long-term outcomes (55, 67, 68). A second theme that was common among participants was a sense of connection to loved ones and ancestors, with several participants additionally remarking on connections to nature and the universe more broadly. Although rarely reported in clinical trials, experiences of connecting with ancestors have been anecdotally noted in the psychedelic community (69) and are common in indigenous psychedelic ceremonies. In a trial of PAT for alcohol dependence, one participant had struggled with alcohol use since her mother's death approximately two decades prior (70). During the dosing session, she “reported to her guides that she saw her deceased mother present in the room with her standing to her left.” Such experiences could potentially be opportunities to process previous traumas and estranged relationships, but further research is needed to delineate the nuances and ethics of such work. Many participants also remarked on a generalized sense of increased gratitude during their dosing sessions, which is consistent with previous research noting enduring increases in the subjective emotion of gratitude following psychedelic experiences (64, 71, 72). Finally, many of these themes align with a qualitative study of people who had self-medicated with psychedelics, which found that reported pain scores improved acutely and after taking psychedelics, with positive reframing and somatic presence playing important roles in improvement (35).

### 4.1 Limitations

Although promising, these findings are limited by a number of important factors which must be addressed with future research. First, our study design did not use a model of PAT that was clinically translational given the large time commitment associated with therapy (e.g., two therapists present during dosing), so our safety results may not translate to larger studies with less psychotherapeutic support or clinical settings. Second, our results are limited by a small sample size that likely does not generalize to typical clinical populations of fibromyalgia given the rigorous screening process that excluded a very high proportion of individuals who were seeking this therapy. The small sample size also means that the findings should be considered as highly preliminary. Third, we had no control group and did not blind study participants or study staff to the treatments administered, which may have affected outcomes given the strong societal narratives around positive treatment outcomes associated with PAT (73). Lastly, further research is needed to validate and explore the role of psychotherapy in the safety and potential efficacy of this treatment (74). The preliminary findings from our small, unblinded study will soon be added to by three other ongoing clinical trials of PAT for FM being conducted elsewhere (Table 5).

**Table 5:** Other clinical trials of psilocybin-assisted therapy for fibromyalgia.

Study title	Sponsor	Study design	*N*	Primary outcome
Psilocybin in patients with fibromyalgia: EEG-measured brain biomarkers of action NCT05548075	Imperial College London	Mechanistic, within-subjects design. 2 doses of up to 25 mg psilocybin with psychotherapeutic support	20	1. Lempel-Ziv complexity 2. The brief experiential avoidance questionnaire
Psilocybin-facilitated treatment for chronic pain NCT05068791	University of Alabama at Birmingham	Parallel group design, double-blind, placebo controlled. 0.36 mg psilocybin/kg body weight (active) 2.5 mg dextromethorphan/kg body weight (placebo)	30	1. Change in daily self-reported pain severity (0–100 visual analog scale)
The impact of psilocybin on pain in fibromyalgia patients (PsiloFM) NCT06368492	Maastricht University	Double-blind, randomized, placebo-controlled. All participants receive doses of placebo, 5 mg psilocybin, 10 mg psilocybin	35	1. Ischemic pain perception 2. Pressure-evoked pain perception 3. Self-reported pain (0–10 visual analog scale)

### 4.2 Conclusions

In this small, open-label clinical trial, we show that PAT was safe and well-tolerated among people with FM, and that individuals generally reported positive impacts on global symptoms and across many FM-related symptom domains. Some participants reported clinically meaningful improvements in pain severity, pain interference, anxiety, and sleep disturbance, with small improvements in chronic pain acceptance. Nonetheless, given the study limitations, larger, controlled studies with a more clinically translational design are necessary to understand whether this therapy is safe and effective in the treatment of FM.

## Data Availability

The datasets presented in this article are not readily available because of the personalized and sensitive nature of the collected data. Requests to access the datasets should be directed to kboehnke@umich.edu.
