# Supplementary material for: Preliminary safety and effectiveness of psilocybin-assisted therapy in adults with fibromyalgia: an open-label pilot clinical trial
Source: Front Pain Res (Lausanne). 2025 Mar 18;6:1527783. doi: 10.3389/fpain.2025.1527783 (PMC11958999; doi:10.3389/fpain.2025.1527783)
Supplement: Supplementary file 1 [file Table1.docx]

|  | **001** | | **002** | | **003** | | **004** | | **005** | | **Average** | |
| --- | --- | --- | --- | --- | --- | --- | --- | --- | --- | --- | --- | --- |
|  | *Pre* | *Post* | *Pre* | *Post* | *Pre* | *Post* | *Pre* | *Post* | *Pre* | *Post* | *Pre* | *Post* |
| **FM Survey score** | 17.0 | 4.0 | 11.0 | 6.0 | 19.0 | 6.0 | 21.0 | 8.0 | 12.0 | 13.0 | 14.6 | 10.4 |
| **Chronic pain acceptance** | 31.0 | 35.0 | 31.0 | 30.0 | 25.0 | 30.0 | 30.0 | 31.0 | 23.0 | 26.0 | 28.4 | 30.4 |
| **Sleep disturbance** | 58.3 | 52.2 | 55.3 | 47.9 | 62.6 | 47.9 | 50.1 | 50.1 | 56.3 | 55.3 | 58.2 | 51.1 |
| **Physical function** | 36.7 | 57.0 | 48.3 | 45.5 | 40.5 | 45.5 | 43.5 | 45.5 | 41.9 | 43.5 | 41.6 | 47.0 |
| **Anxiety** | 51.2 | 40.3 | 55.8 | 48.0 | 55.8 | 48.0 | 51.2 | 48.0 | 55.8 | 51.2 | 53.3 | 47.7 |
| **Depression** | 41.0 | 41.0 | 41.0 | 41.0 | 51.8 | 41.0 | 55.7 | 41.0 | 53.9 | 49.0 | 45.7 | 45.5 |
| **Fatigue** | 58.8 | 51.0 | 53.1 | 48.6 | 64.6 | 48.6 | 55.1 | 57.0 | 62.7 | 62.7 | 60.8 | 54.9 |
| **Cognitive abilities** | 44.3 | 54.7 | 50.5 | 50.5 | 44.3 | 50.5 | 50.5 | 50.5 | 44.3 | 44.3 | 45.5 | 50.1 |
| **Pain Interference** | 66.6 | 41.6 | 57.1 | 55.6 | 63.8 | 55.6 | 57.1 | 53.9 | 61.2 | 57.1 | 62.5 | 53.1 |
| **Pain severity** | 5.9 | 2.0 | 4.0 | 1.1 | 5.4 | 1.1 | 4.4 | 2.4 | 3.7 | 2.7 | 4.9 | 2.5 |

Table 1. CPAQ: Chronic Pain Acceptance Questionnaire. Pain Severity reported as change in aggregate pain score from the 7 days prior to the intervention to the end of the intervention. Sleep disturbance, pain interference, physical function, anxiety, depression, fatigue, participation in social activities, and cognitive abilities are all reported as T-scores per PROMIS scoring. Negative change scores indicate improvement for pain severity, pain interference, sleep disturbance, FM score, anxiety, depression, and fatigue. Positive change scores indicate improvement for CPAQ, physical function, participation in social activities, and cognitive abilities.

**Appendix table 1. Participant scores for each measure.**
